# Remarks on the Use of Clasps, (for the Retention of Artificial Teeth)

**Published:** 1850-04

**Authors:** P. H. Austen

**Affiliations:** Baltimore


From the American Journal of Dental Science.

### REMARKS ON THE USE OF CLASPS, (FOR THE RETENTION OF ARTIFICIAL TEETH.)

BY P. H. AUSTEN, M. D., OF BALTIMORE.

*Messrs. Editors:*

WE should be glad to see some remarks, from the pen of any of the experienced members of the profession, upon the follewing question: How far may the principle of Cleveland's air cavity be substituted for clasps in the retention of partial pieces of plate work? The question is one of eminent practical importance, and as such, its discussion deserves a place in a journal devoted to the improvement of dental science.

This subject has been suggested to our own mind by the obvious and acknowledged objections which lie against the use of clasps in many, (may we not say in all?) cases of their application. A concise exposition of some of these objections may place in a clearer light the importance of the query above propounded.

In the first place, the choice of the teeth to which clasps may be attached is limited. No judicious operator will clasp around the incisores or cuspidati for the retention of a single tooth, much less a piece of several teeth. The clasp in this situation will be more or less visible—a serious objection with the patient, and one which sometimes holds good when a first biscuspis is chosen as the tooth to be clasped. The shape of these teeth opposes the retention of clasps, unless placed so far up upon the tooth, as almost inevitably to cause irritation and periosteal inflammation. Lastly, these teeth are more liable than the molares, to have their position deranged, either in a lateral or veitical direction, by the traction necessarily exercised by the artificial piece through the medium of its clasps. It is a well known physiological fact, one upon which depends our ability to correct irregular dentures—that a very moderate force, if continuously applied, is sufficient to change the position of a tooth; on the other hand, there unquestionbly is such force exerted, moderate though it be, in the retention of every piece, tending either to elongate the teeth, especially where they have no antagonist, or to change their position laterally; and in just so far deranged the adaptation of the plate.

Again, with respect to the choice of the teeth, there is this objection to the use of the third molares, that unless the plate can be allowed to extend to some distance behind them, which is rarely ever admissible, the weight of the piece, being altogether in front, acts with an undue leverage upon the teeth, and brings a strain upon them which must ultimately result in their loss. In the case of the other molares or of the biscuspides, where the plate can extend behind as well as before the line of the teeth clasped, the traction is more immediately vertical, and, where there are teeth below to antagonise, is less likely to prove hurtful.

If, in addition to the above remarks, we take the fact of the greater liability of the first molar to loss from caries or other causes—great in the proportion of two to one than in the case of the second molares or the second biscuspis, and in a still higher proportion compared with the other teeth—we shall find that the number of teeth are limited, to which clasps may be attached with comparative safety. We shall often meet with cases where, in the absence of suitable teeth, we must attach to those liable to the above named objections, or else adopt the other alternative, (to which we should gain a very reluctant consent from our patient,) and extract them with a view to supply a complete upper set. The difficulty has, in a few instances, been overcome by the exact adaptation of a plate over the whole palatine arch, being careful that the edge of the plate shall not come too closely against the necks of the teeth still remaining in the mouth. Will not the same purpose be better answered and with less chance of failure by the use of a smaller plate than Cleveland's air chamber.

We have alluded to the physiological principle upon which is based the success of operations, for the regulation of teeth. This success is limited to a certain age, varying in different individuals, from eighteen to twenty-five. After this period, the same force which could with impunity be applied, will excite periosteal inflammation, and cause the loss of the tooth. The same result often attends the separation of teeth by means of cotton, wood or india rubber, preparatory to filling cavities on their approximal surfaces ; and precisely such a result must sometimes happen to teeth to which clasps are attached, whether incisores, cuspidati, biscuspides or molares. This forms our second objection, most applicable to the incisors and cuspidati, but also in some degree to the biscuspides and molares.

A third difficulty is the retention of vitiated mucus or other fluids in the mouth, between the clasp and the tooth. This will take place, however closely the clasp may be adapted, and will act more or less injuriously upon the tooth, however careful the patient may be to cleanse the piece daily. Especially is this liable to occur where, from the presence of decay, or other causes, the file has been used. It is the practice of many to file a badly shaped tooth so as better to adapt the clasp; also, with the same instrument to separate two teeth to allow the clasp to pass between them It is unnecessary to observe that such practice materially affects the permanence of the piece by increasing the loss of the tooth thus treated. We would not be understood as censuring those who adopt this course—it is frequently impossible to do otherwise ; but wherever in our own practice, such a necessity for the use of the file should occur, we would anxiously inquire if the piece might not be retained in the mouth in some other way than by clasps.

The biscuspides and molares of many persons have crowns so short, or else of so conical a form—larger at the neck than at the base, or *vice versa*—that the adjustment and retention of clasps is exceedingly difficult, and sometimes, unless the file is resorted to, impossible. Again, the close adaptation of the plate and clasp around the neck of the tooth, is not unfrequently a couse of irritation, and subsequent inflammation of the dental and alveolar periosteum. This, like a previous objection, holds with more force against the single than against the double fanged teeth. Yet in cases of peculiar susceptibility, such as are by no means uncommon, it will apply to all. It is to avoid this danger, that the anterior margin of a plate must not be carried too close to such front teeth as may be still remaining. Some dentists seek to obviate the well known difficulty, by soldering a piece of half-round wire to the plate, thus giving it a thicker edge, at the same time bringing it to the very margin of the gum—a very useful and effective measure in case of a *lower* piece, to guard the softer parts against irritation by the otherwise too short border; but not so suitable in the present case, because it is not the gum which is irritated so much as the neck of the tooth itself, by the constant pressure, gentle though it may be, of an unyielding metalic plate.

The above remarks are made, not in unreserved condemnation of the use of clasps, but rather as suggestive of the imperfection of the method. However excusable or necessary in any given case, it is, under the most favorable circumstances, an *imperfect* method—imperfect because it endangers sound natural teeth, which are very preferable to the best artificial substitutes—imperfect, because it violates fixed physiological principles, and if harmless, is only so by a species of courteous forbearance on the part of nature.

Our art has made rapid improvement, is every day improveing, must still greatly improve ere it reaches perfection. What we consider now as defective, is still an improvement on the past; nor should we condemn and discard any plan till we have found a more unobjectionable substitute. To inquire after such a substitute, is the design of this article. Admitting that there are here and there cases where clasps may be most conveniently applied, aud worn for years without injury, we ask—may not the air-chamber or suction plate be substituted, in the great majority of cases, with decided benefit?

Of its size, shape and position, in particular cases, we are not at present prepared to speak—judgment and experience must guide the operator. We think the Journal might be made the medium of much valuable information on this point.

Among the objections to its use, we would briefly notice: First, the interference with distinct articulation, occasioned by the prominence of the plate in the palatine arch. With many this is an important consideration, and if the impediment be considerable and permanent, will prevent the adoption of the plan. Again, the portion of membrane drawn into the opening of the cavity, becomes sometimes exceedingly tender and sensitive. This, if persistent, will also prevent the substitution of such a piece, for one with clasps. Lastly, the additional expense: it requires more gold, and takes more of that which, to the professional man, is of more value than gold—his time. These objections we cannot do more at present than simply name. Upon the last of the three, we could wish to dwell more at length, not only upon its bearing in this one point, but also as connected with professional services generally; we hope on some future occasion to present to your consideration, some remarks upon the "character of dental operations, and their due compensation."

If the above remarks should elicit from any of your numerous readers, the detailed result of their individual experience, touching the question proposed, they will fully have answered, Messrs. Editors, the purpose for which they were written.

P. H. AUSTEN.

---

MEDICAL TESTIMONY.—Such is the exactness of the evidence given on Prof. Webster's trial, by medical gentlemen of Boston and vicinity, that the community must be convinced of the profound attainments of those who are thus shown to be masters of their profession. The re-construction of the skeleton of the late Dr. Parkman, by the mere fragments of different bones which were found, indicates the very deep study that has been bestowed on anatomy by those who were able to accomplish a feat so novel and remarkable. Dr. Keep's careful and explicit testimony, too, shows what perfection the art of dentistry has attained in New England, and exhibits in an interesting manner the mode of making records by moulds of the parts to be benefited by art. The silent evidence of a mineral tooth, sliding into the matrix in which it was made, and fitting no other place, amounts to demonstration in a case like this. There is a kind of medical testimony that is weakened by efforts to give it strength: those who venture beyond what is requisite, by advancing hypotheses, instead of keeping close to the lines drawn by nature and fact, add nothing to the credit of the profession, and do not conform to what is required by modern medical jurisprudence. Little of this has been manifested, however, on the present exciting trial.—*Bost. Med. and Surg. Journal.*

---

Prof. Webster's Conviction.—The trial of Dr. Webster, which has occupied the public mind for the last fortnight to an extent unprecedented in a criminal case, was terminated late on Saturday evening last by a verdict of *guilty* from the jury. Notwithstanding the general sentiment, from a careful analysis of the testimony during the trial, that the prisoner was guilty of the awful crime for which he was indicted, the announcement of the verdict produced a shock, which was felt through the whole city. Thus a man who has been in the highest walks of society, esteemed one of the pillars of science, and a professor in Harvard University, the first and oldest institution of learning in America, must suffer death, for the horrible crime of murdering a fellow being—a member of the same profession with himself. The trial may be considered as one of the most important on record, and possesses a painful interest to the medical profession. In addition to the government testimony which has already been given in the Journal, it may be well to state in a few words that the janitor of the College testified to an interview on Monday, the 19th, at the College, between Drs. Parkman and Webster, in which loud words were used by the former respecting the money due him; that an appointment of another meeting, on the fatal Friday succeeding, was made by Dr. W., which he himself acknowledges, and also says that Dr. P. came and he paid him some money; that Dr. W.'s rooms were kept constantly locked after that day, unusual heat was kept up in the furnace, and that tan and faggots were brought to the College; that the twine found around the remains corresponded with a ball in Dr. W.'s room; and that anonymous letters sent to the City Marshal, directing his attention, in searching for the remains, away from the College, were thought by experts in penmanship to have been written by Dr. W. The defence consisted mainly in the testimony of various highly respectable individuals that the character of Dr. W. was good as a quiet and peaceable man—of his three daughters as to the time spent at home by their father before his arrest—of four or five individuals that they saw Dr. Parkman after 2 o'clock on Friday—and of Dr. Morton respecting the teeth found in the furnace, which will be found in another part of to-day's Journal. Sentence of death was passed upon Prof. Webster on Monday—the day of execution to be appointed by the Governor.—*Bost. Med. and Surg. Jour., April 3d.*

